# Evolution of Thermal Plasticity in *Hymenoscyphus fraxineus* During Ash Dieback Expansion in Europe

**DOI:** 10.1002/ece3.71513

**Published:** 2025-06-17

**Authors:** Clémence Bécans, Cécile Robin, Katharina B. Budde, Luisa Ghelardini, Andrin Gross, Vaidotas Lygis, Lene Rostgaard Nielsen, Gilles Saint‐Jean, Jean‐Paul Soularue

**Affiliations:** ^1^ Univ. Bordeaux, INRAe, UMR1202 BioGeCo Cestas Cedex France; ^2^ Northwest German Forest Research Institute Hann Muenden Germany; ^3^ Department of Agricultural, Food, Environmental and Forestry Science and Technology (DAGRI) University of Florence Firenze Italy; ^4^ Swiss Federal Institute for Forest, Snow and Landscape Research WSL, Biodiversity and Conservation biology Birmensdorf Switzerland; ^5^ State Scientific Research Institute Nature Research Centre Vilnius Lithuania; ^6^ Department of Geosciences and Natural Resource Management University of Copenhagen Frederiksberg C Denmark

**Keywords:** ash dieback, *Chalara fraxinea*, *Hymenoscyphus fraxineus*, *Hymenoscyphus pseudoalbidus*, invasive fungal pathogen, microbial evolution, plasticity evolution, thermal plasticity

## Abstract

The plasticity of adaptive traits may be critical for population persistence in heterogeneous environments. However, its evolution is rarely investigated in forest pathogens, potentially limiting the accuracy of epidemic risk predictions. Ash dieback is an emblematic example of a forest epidemic caused by an invasive fungal pathogen—*Hymenoscyphus fraxineus*, which has likely been introduced to Eastern Europe from East Asia. We investigated the plasticity and thermal niche evolution of *H. fraxineus* during its spread across Europe. We characterized the reaction norms of in vitro mycelial growth and viability of *H. fraxineus* isolates from five European populations sampled along a latitudinal gradient spanning from Lithuania to Italy. While all populations responded uniformly to temperature decrease, their responses to temperature increase diverged markedly. The growth of *H. fraxineus* isolates from the northernmost population (Lithuania) was most negatively affected by high temperatures, whereas the southernmost isolates (Italy) showed optimal growth at a higher temperature compared to the other populations. Additionally, the viability of Lithuanian isolates was significantly reduced by higher temperatures compared to that of the other populations. These findings suggest that both growth plasticity and thermal niche have evolved during the pathogen's expansion in Europe, with potentially important implications for predicting and managing future epidemic risks. We further discuss how evolutionary processes may have shaped these phenotypic differences.

## Graphical abstract

We investigated the evolution of phenotypic plasticity in *Hymenoscyphus fraxineus*, the fungal pathogen responsible for ash dieback epidemics across Europe. To this end, we collected samples from five European populations along a latitudinal gradient, ranging from Lithuania to Italy. Our results reveal significant differences in thermal reaction norms for growth and viability among these populations, suggesting that both growth plasticity and the thermal niche of *H. fraxineus* have evolved during the pathogen's expansion across Europe.

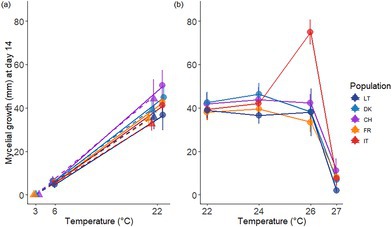

## 1 Introduction

Phenotypic plasticity refers to the influence of the environment on phenotypic expression (DeWitt and Scheiner 2004). Unlike genetic evolution, which requires successive generations to induce phenotypic changes in a population, phenotypic plasticity allows a genotype to express varying phenotypes in response to a changing environment within the lifetime of an individual. Therefore, phenotypic plasticity can play a crucial role in the expansion and persistence of populations facing unfavorable environmental conditions (Bouteiller et al. 2021; Gomulkiewicz and Stinchcombe 2022; Kirkpatrick and Barton 1997; Scheiner et al. 2020). However, the diversity of the plastic responses of populations and their impact on fitness remain elusive in numerous species, especially in microbes (Alster et al. 2021). One of the major reasons for this is that phenotypic plasticity itself can be genetically variable and evolve along highly context‐dependent trajectories (Lande 2015; Reed et al. 2010; Scheiner et al. 2012; Soularue et al. 2023). In addition, strong demographic fluctuations in microbial populations make it difficult to apply theory to real‐world situations, such as range expansion, introduction into new areas, or landscape fragmentation (Engen et al. 2013; Leung et al. 2020; Xue and Leibler 2018). Empirical studies of phenotypic plasticity and its evolution are therefore essential for anticipating population dynamics (Bacigalupe et al. 2018; Bebber and Chaloner 2022; Tong et al. 2023) and for implementing effective ecosystem management programs (Chevin et al. 2010; Forsman 2015; Gomulkiewicz and Stinchcombe 2022).

Ash dieback is an emerging and lethal disease affecting European ash species, of which the most susceptible are common ash ( *Fraxinus excelsior* L.) and narrow‐leaved ash ( *F. angustifolia* Vahl) (Schwanda and Kirisits 2016). The disease is caused by *Hymenoscyphus fraxineus* (T. Kowalski) Baral, Queloz & Hosoya, an invasive ascomycetous fungal pathogen. *H. fraxineus* likely originates from East Asia, where it is associated with Manchurian ash ( *F. mandshurica* Rupr.), as a latent endophyte or foliar pathogen (Cleary et al. 2016; Zhao et al. 2013). This inconspicuous lifestyle of the pathogen has probably led to its introduction in Europe with Manchurian ash trees (Drenkhan et al. 2014). Genomic studies indicate that *H. fraxineus* may have been introduced 14–32 years before its massive invasion across Europe began during the early 1990s (Sønstebø et al. 2017). This timeline was further supported by the detection of *H. fraxineus* in Estonian herbarium samples of Chinese ash ( *F. chinensis* Roxb.) trees dated to as early as 1978 (Agan et al. 2023). Following what appears to have been a single introduction event (McMullan et al. 2018; Gross, Hosoya, and Queloz 2014), *H. fraxineus* has spread rapidly across Europe, successfully establishing itself in regions where susceptible ash species are prevalent. The first symptoms of ash dieback were observed in Poland in 1992 (Timmermann et al. 2011); the epidemic reached northeastern France in 2008 (Ioos et al. 2009) and northeastern Italy one year later (Ogris et al. 2010). More recently, the fungus has also been detected in Spain (Stroheker et al. 2021). *H. fraxineus* has an annual life cycle. It overwinters in leaf litter and produces apothecia on the rachises of decomposing ash leaves through heterothallic sexual reproduction (Gross, Holdenrieder, et al. 2014). During summer, ascospores are wind‐dispersed (Grosdidier et al. 2018; Timmermann et al. 2011) and cause new infections upon contact with living ash leaves. The very high mortality rates of native European ashes (> 80%) observed in some areas (Coker et al. 2019; Husson et al. 2012; McKinney et al. 2014) constitute a major threat not only to the European ash trees but also to the biodiversity associated with them (Hultberg et al. 2020; Mitchell et al. 2014; Pautasso et al. 2013).

Temperature is a strong determinant of the geographical distribution of species (Arnold et al. 2024; Bacigalupe et al. 2018). This is particularly true for ectotherms like fungi that show limited ability to regulate their own temperature level (Desprez‐Loustau et al. 2007; Gerken et al. 2015; McLean et al. 2005; but see Cordero et al. 2023). The rapid latitudinal spread of the ash dieback epidemic in Europe suggests a substantial ability of *H. fraxineus* to mitigate thermal stress, thus exhibiting high thermal tolerance. The evolution of phenotypic plasticity may have facilitated *H. fraxineus*' resilience to temperature variations, particularly at the edges of the species distribution range where environmental conditions can be extreme (Benito Garzón et al. 2019; Gallet et al. 2014; Gilchrist 1996). Thermal tolerance is often described by ecologists with thermal performance curves that represent the relationship between an organism's performance and temperature (Fordham et al. 2013). Interestingly, there is a tight link between the thermal performance curves and thermal reaction norms, describing the way adaptive traits vary across a range of temperatures (Chevin et al. 2010; Kingsolver and Huey 2008; Lande 2015; Levins 1968). Such reaction norms are usually unimodal and allow identification of the temperature at which trait expression is optimal, as well as critical temperatures hindering phenotypic expression (Bacigalupe et al. 2018). Thereby, characterizing the thermal plasticity of fitness‐related traits in *H. fraxineus* populations can help to characterize the thermal limits of the pathogen, the optimal temperature for its development, and the evolution of its thermal reaction.

Kowalski and Bartnik (2010) and later Hauptman et al. (2013) characterized the in vitro mycelial growth of *H. fraxineus* at different temperatures. This trait is considered adaptive in plant pathogenic fungi as its expression likely affects their ability to infect and colonize their hosts (Blenis et al. 1984; Kowalski and Bartnik 2010). As observed for other fungi (e.g., some chitinolytic ascomycetes—see McLean et al. 2005; *Cryphonectria parasitica* (Murrill) M.E. Barr—see Robin et al. 2017), the influence of temperature on in vitro growth rate was isolate‐dependent. Assuming that the investigated *H. fraxineus* isolates differed genetically, this suggests that the thermal plasticity of mycelial growth is genetically variable and can evolve. Kowalski and Bartnik (2010) and Hauptman et al. (2013) also determined an optimal temperature for in vitro mycelial growth of *H. fraxineus*, which ranged from 20°C to 24°C. Low temperatures (5°C) constrained but did not prevent mycelial growth, while high temperatures (30°C) almost systematically prevented it. In addition, Hauptman et al. (2013) showed that the viability of *H. fraxineus* in ash tissues was very low after a five‐hour exposure to hot water (36°C) treatment. More recently, in situ observations in France coupled with statistical modeling showed that ash dieback severity was influenced, among other things, by climatic conditions (Grosdidier et al. 2020; Marçais et al. 2023). Yet, none of these studies addressed the evolution of phenotypic plasticity.

The aim of the present study was to determine whether the thermal reaction norms for mycelial growth and viability of *H. fraxineus* have evolved during the spread of the disease from the putative center of the epidemic in eastern Europe toward southern Europe. To address this question, we examined the in vitro growth and viability at different temperatures of replicated isolates from five European *H. fraxineus* populations sampled along a latitudinal gradient. The contrasting temperature regimes among the sampling locations (Lithuania, Denmark, Switzerland, France, and Italy) likely imposed divergent selective pressures on European populations of the pathogen (Booker 2024; Siepielski et al. 2013), potentially driving differentiation in growth and viability reaction norms (Schmid et al. 2019; Valladares et al. 2014), along with distinct thermal preferences and limits (Bacigalupe et al. 2018; Ruiz‐Aravena et al. 2014). Furthermore, the fungus likely underwent successive founder effects during its spread (Arnaud‐Haond et al. 2006; Peter and Slatkin 2015), potentially influencing reaction norm variability along the latitudinal gradient (Blanquart et al. 2012; Robertson 1960). Therefore, we specifically tested three main hypotheses:

1. The thermal reaction norms for growth and viability differ between European populations of *H. fraxineus*.
2. More specifically, *H. fraxineus* isolates representing populations from southern Europe exhibit better growth and viability at higher temperatures compared to northern populations of the fungus, along with a shift in their thermal limits.
3. As a consequence of natural selection and genetic drift, a spatial structure in the intra‐population variability of the reaction norm characteristics is expected along the latitudinal gradient.

## 2 Materials and Methods

### 2.1 Tested Populations of *Hymenoscyphus fraxineus*

In order to study *H. fraxineus* populations with different epidemiological histories, we selected populations established at varying distances from the initial outbreak site of ash dieback in Europe (Poland), specifically in Lithuania, Denmark, Switzerland, France, and Italy (Table 1). The sampling spanned from locations near the northernmost emergence site of the disease (Lithuania) to locations near the southern front of the epidemics (Italy, France), where summer temperatures frequently exceed previously proposed thermal limits in *H. fraxineus* (Ghelardini et al. 2017; Hauptman et al. 2013; Kowalski and Bartnik 2010; Luchi et al. 2016; Stroheker et al. 2021). The populations ranged, at the time of sampling, from the oldest established in Lithuania to the most recently established in Italy, thus representing the distribution of the fungus in Europe. Importantly, based on average summer and winter temperatures estimated by WorldClim (version 2, Fick and Hijmans 2017), the sampled populations experienced diverse climatic conditions (Table 1). Rachises from the senesced leaves of *F. excelsior* from previous years, exhibiting symptoms of infection by *H. fraxineus* (Gross, Holdenrieder, et al. 2014), were randomly collected between July 4, 2020, and August 11, 2020, at the base of multiple trees at each location. Upon collection, the samples were sent to France (Biogeco, INRAE Bordeaux) and stored at 4°C until the isolation procedure (realized from the 17th to the 19th of September 2020). The isolation protocol followed Kowalski and Bartnik (2010) with modifications: the rachises were sterilized using 96% ethanol, and the medium utilized for fungal isolation and culturing was Ash Leaf Malt Extract Agar (AMEA, Kirisits et al. 2013). From each population, 15 isolates showing the morphological characteristics of *H. fraxineus* were randomly selected, each from a different rachis. In total, 75 isolates were subsequently stored in a cold room at 3°C for preservation. To verify the species identity of isolates showing singular morphotypes, DNA was extracted from the obtained isolates, and the internal transcribed spacer (ITS) region of the nuclear ribosome was sequenced. The sequences were compared with publicly available sequences of *H. fraxineus* in the National Center for Biotechnology Information (NCBI; https://blast.ncbi.nlm.nih.gov/Blast.cgi) database with the BLAST algorithm. Each ash tree can be infected by multiple *H. fraxineus* genotypes, and the fungus reproduces sexually (Landolt et al. 2016). As population genetics studies have always shown a high genotypic diversity (Burokiene et al. 2015; Gross et al. 2012; Gross, Hosoya, and Queloz 2014; Kraj et al. 2012), the isolates we studied were likely genetically distinct from each other (Junker et al. 2017). Before each experiment described herein, each isolate was subcultured on a new AMEA plate and incubated for three weeks at 22°C under a controlled day/night cycle (16 h light/8 h dark).

**TABLE 1:** GPS coordinates and elevation (m) of sampled *Hymenoscyphus fraxineus* populations, along with mean annual temperature (°C) and precipitation (mm) at each site estimated by Worldclim for the years 1970–2000 (v2, Fick and Hijmans 2017).

Population	Country	Coordinates	Elevation	Temperature	Precipitation	Year of first report
LT	Lithuania	54.8284 N 24.1013 E	62	6.58	607	1996
DK	Denmark	55.8955 N 12.4579 E	28	8.00	601	2002
CH	Switzerland	47.3563 N 8.4543 E	529	8.96	1160	2008
FR	France	44.3539 N 2.5378 E	562	10.82	740	2019
IT	Italy	44.0514 N 10.8430 E	844	9.71	1873	2016

*Note:* The last column indicates the year of the first report of ash dieback disease at the sampling site.

### 2.2 Effect of Temperature on In Vitro Mycelial Growth of *H. fraxineus*

We assessed the mycelial growth of the *H. fraxineus* individuals across a range of constant temperature treatments: 3°C, 6°C, 22°C (control), 24°C, 26°C, and 27°C. This range encompassed both sub‐optimal and supra‐optimal temperatures relative to the optimal growth temperature of 22°C identified by Hauptman et al. (2013). For each individual, we initiated cultures by placing a 5 mm diameter agar plug overgrown with mycelium of *H. fraxineus* taken from the margin of the fungal cultures at the center of the Petri plates (85 mm), each containing 15 mL of AMEA medium. Three replicate plates per individual were incubated at each temperature treatment in the dark for two weeks. After 14 days of incubation, the colony diameter was determined as the average of the longest and the shortest diameters (two measurements). Therefore, the diameter of each colony at a single time point was used as a proxy for mycelial growth. For technical reasons, the study was segmented into three distinct experiments, each including the 22°C control temperature treatment. The effect of higher temperatures (24°C, 26°C, and 27°C) on isolate growth was examined in November 2020, this experiment is referred to as the “high temperatures growth experiment.” The other two experiments focused on lower temperatures: one testing the effect of a 6°C treatment in March 2023, referred to as the “6°C growth experiment,” and another testing a 3°C treatment in June 2023, referred to as the “3°C growth experiment.”

### 2.3 Effect of Temperature on the Viability of *H. fraxineus*

We evaluated the viability of each individual after 10 days of exposure to low (−20°C) or high temperatures (30°C–32°C) by examining the proportion of agar plugs that resumed growth at the control temperature (i.e., 22°C sensu Hauptman et al. 2013). The scale from 0 to 1 was used, where 0 means that no agar plug resumed growth following treatment, and 1 means that 100% of the plugs resumed growth. This part of the study consisted of two distinct experiments conducted in February 2022 (30°C–32°C) and May 2023 (−20°C). Both experiments utilized 5 mm diameter agar plugs overgrown with *H. fraxineus* mycelium, which were taken from the margins of 3‐week‐old isolate cultures. For the high‐temperature treatments, nine agar plugs per isolate and per temperature treatment were placed in a common plate containing AMEA for 10 days at 22°C (control), 30°C, 31°C, and 32°C. On the 11th day, these plates were incubated at 22°C for three weeks, during which we visually assessed the resumption of mycelium growth from each agar plug. The absence of growth after three weeks was considered a non‐viable propagule.

For the low‐temperature treatment (May 2023), nine agar plugs per isolate and per temperature treatment were placed in 2‐mL Eppendorf tubes and exposed to −20°C and 22°C (control) temperatures for 10 days. On the 11th day, each plug was transferred from its tube to a new common plate containing 15 mL of AMEA medium and then incubated at 22°C. We monitored the viability of the agar plugs over a 24‐day period.

### 2.4 Statistical Analyses

Our experiments generated five distinct datasets, each related to mycelial growth or viability as a function of temperature. We independently analyzed each dataset using linear and generalized mixed models in R software (v4.3.3; R Core Team 2024). Data handling was carried out using the Tidyverse package (v2.0.0, Wickham et al. 2019), model fitting was done with the lme4 package (v1.1‐35.4, Bates et al. 2015), and model predictions were obtained with the stats package (v4.4.1; R Core Team 2024).

We estimated linear reaction norms at the isolate level. Isolate reaction norms were characterized by an intercept (trait value in the reference environmental conditions, i.e., at 22°C) and a slope (change in trait expression in relation to temperature, also referred to as the level of plasticity) (Hendry 2016; Soularue et al. 2023). Hence, when a dataset comprised more than two temperature treatments, we divided the overall reaction norm of each *H. fraxineus* isolate into linear segments. These segments linked a pair of trait values: one observed under control temperature (22°C) and the other observed under varying temperature treatments, presumed to be non‐optimal for *H. fraxineus* (Hauptman et al. 2013). Our aim was to facilitate the interpretation of the parameters of the models fitted to the datasets. To improve the goodness of model fitting and limit model convergence issues, we specified temperature as a fixed effect and considered the interaction effect between temperature and populations and between temperature and isolates as random (see examples given by Bolker et al. (2008) and Arnold et al. (2019)). The “isolate” effect was nested within the “population” effect. We checked the goodness of fit of our models using marginal and conditional *R* ^2^ (MuMIn R package, v1.48.4, Bartoń (2024)) and visual comparisons of model predictions against the data. Sometimes the variance estimates for isolated random effects were very low. In such cases, when possible, cross‐validation using simpler models without isolated effects was performed during preliminary analyses.

We tested our three hypotheses by using the following model:

(1) $$Y_{\mathrm{ijk}}=\beta_{0}+b_{0j}+\gamma_{0\mathrm{ij}}+\left(\beta_{1}+b_{1j}+\gamma_{1\mathrm{ij}}\right)e_{\mathrm{ijk}}+\epsilon_{\mathrm{ijk}}$$

where $Y_{\mathrm{ijk}}$ represents either fungal colony diameter (in mm) or viability of replicate k of isolate i belonging to population j. In the case of viability, $Y_{\mathrm{ijk}}$ followed a Bernoulli law and was replaced in (1) by log($\pi_{\mathrm{ijk}}/1-\pi_{\mathrm{ijk}}$), where $\pi_{\mathrm{ijk}}$ is the probability that replicate k of a propagule from isolate i in population j is viable. $\beta_{0}$ is the estimated fixed intercept of the response variable Y when the temperature effect e is at its reference value. $\beta_{1}$ is the estimated fixed slope, representing the change in the response variable Y for a one‐degree (°C) variation in temperature. $b_{0j}$ is the estimated random intercept for the population j, representing how much the intercept for population j deviates from the overall average intercept $\beta_{0}$. $b_{1j}$ is the estimated random slope for population j, representing how much population j's slope deviates from the overall average slope $\beta_{1}$. $\gamma_{0\mathrm{ij}}$ and $\gamma_{1\mathrm{ij}}$ represent isolate‐specific deviations of the intercepts and the slope of reaction norms, respectively, the isolate being nested within populations.

We tested our three hypotheses using bootstrapping with the boot package (v1.3‐30, Davison and Hinkley 1997), conducting 1000 iterations for each dataset and hypothesis. Testing of hypothesis 1 focused on population differences in the intercept ($\beta_{0}+b_{0j}+\gamma_{0\mathrm{ij}}$) and slopes ($\beta_{1}+b_{1j}+\gamma_{1\mathrm{ij}}$) of isolate reaction norms. Hypothesis 2 was tested from bootstraps of model (1)'s predictions at each treatment temperature where trait values were non‐zero (6°C–27°C for growth, −20°C to 32°C for viability). Tests of hypothesis 3 determined whether there were population differences in the standard deviation of the intercepts and slopes of isolate reaction norms. For all three hypotheses, we employed a conservative approach: differences between populations were considered significant only when the confidence intervals of the populations' mean estimates did not overlap. The results of model bootstrapping, along with details about the significance of the fixed temperature effect ($\beta_{1}$) and model performance, are summarized for each temperature interval (control and treatment temperatures) in Table 2.

**TABLE 2:** Summary of model (1)' analysis and performance.

Temperature treatment (°C)	Trait	T. effect	Pop. effect on RN		Pop. effect on variability of individual RNs		$R_{m}^{2}$	$R_{c}^{2}$
			Intercept	Slope	Intercept	Slope		
3	Growth	Yes (*p* < 0.001)	No	Yes	Yes	Yes	0.84	0.98
6	Growth	Yes (*p* < 0.001)	No	Yes	Yes	Yes	0.81	0.96
24	Growth	No (*p* = 0.24)	No	Yes	Yes	Yes	0.00	0.62
26	Growth	No (*p* = 0.53)	No	Yes	No	Yes	0.03	0.86
27	Growth	Yes (*p* < 0.001)	No	Yes	No	Yes	0.74	0.91
−20	Viability	No (*p* = 0.32)	No	No	Yes	Yes	0.13	0.97
30	Viability	No (*p* = 0.65)	No	Yes	Yes	Yes	0.00	0.44
31	Viability	No (*p* = 0.19)	No	Yes	Yes	Yes	0.21	0.76
32	Viability	Yes (*p* < 0.001)	No	No	Yes	Yes	0.59	0.96

*Note:* Each individual reaction norm was estimated from replicated trait values observed at the reference temperature (22°C) and under a temperature treatment. T. effect: Fixed effect of temperature on the response variable (isolate diameter or viability), Pop. effect: Population random effect on the response variable. “Yes” or “No” indicates whether the effect is statistically significant at the 0.05 significance level. Individual RNs: Individual reaction norms. $R_{m}^{2}$: Marginal *R* ^2^ representing the proportion of variance in the response variable explained solely by the fixed effects. $R_{c}^{2}$: Conditional *R* ^2^ representing the proportion of variance in the response variable explained by the entire model, including both fixed and random effects.

## 3 Results

### 3.1 No Variation in Growth and Viability Across *H. fraxineus* Populations at the Control Temperature

**FIGURE 1:**
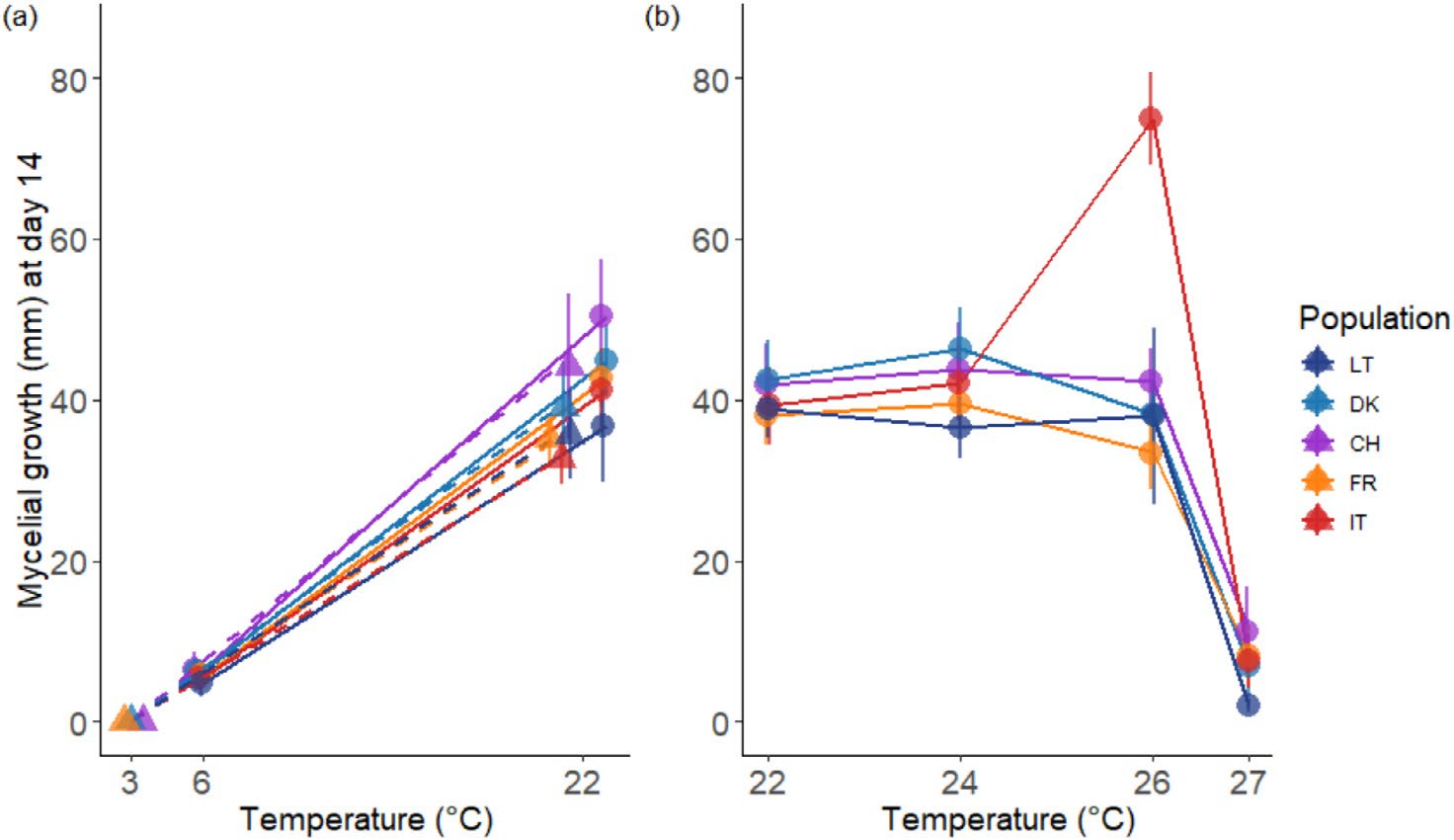
Mycelial growth of *Hymenoscyphus fraxineus* isolates in response to different temperature treatments. Each data point represents the mean fungal culture diameter of 15 isolates within a specific population. For each isolate at each temperature, the diameter value corresponds to the average of three replicates. (a) Results of two different experiments focusing on low temperatures (3°C and 6°C), each with 22°C as a control condition. For clarity, data from the two experiments are not aligned with this value. The 3°C experiment is represented by triangles and dashed lines. The 6°C experiment is represented by circles and solid lines. (b) Results of a single experiment focusing on high temperatures. Error bars indicate the 95% confidence interval of the mean. Colors and letters correspond to the origin of the isolates: Dark blue represents Lithuania (LT), blue represents Denmark (DK), purple represents Switzerland (CH), orange represents France (FR), and red represents Italy (IT).

**FIGURE 2:**
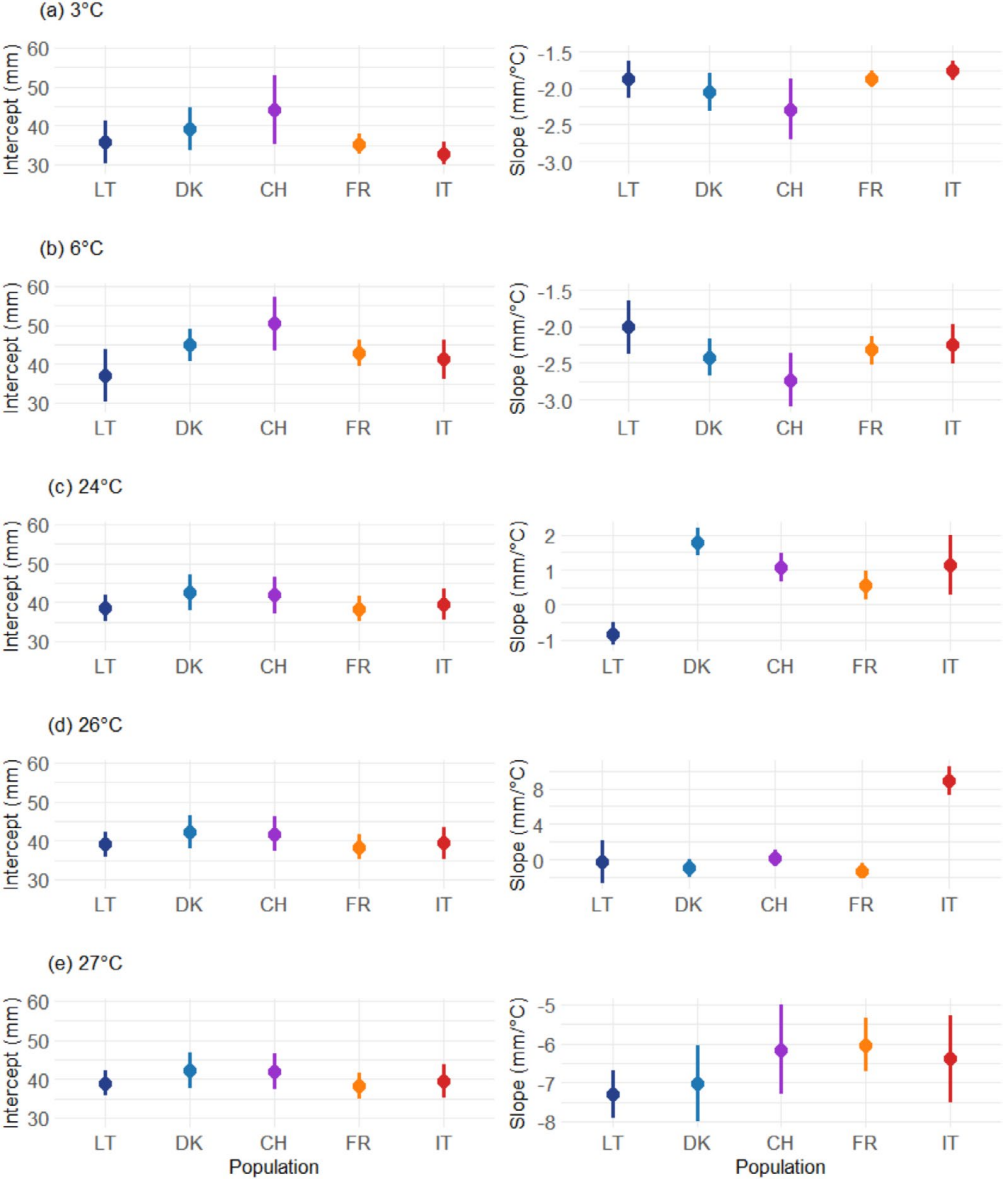
Estimated intercepts (mm) and slopes (mm/°C) of growth reaction norms in five European *Hymenoscyphus fraxineus* populations exposed to different temperatures for two weeks. Panels (a) to (e) correspond to the different temperature treatments. The reaction norms were estimated based on individual diameter measurements at 22°C (control temperature) and at the corresponding treatment temperature. Each point represents the mean estimate of 15 fungal isolates within a specific population, with three replicates per isolate and per temperature. Error bars indicate the 95% confidence intervals of the estimates. Colors and letters correspond to the origin of the isolates: Dark blue represents Lithuania (LT), blue represents Denmark (DK), purple represents Switzerland (CH), orange represents France (FR), and red represents Italy (IT).

At the control temperature of 22°C, the mean measured colony diameters of all tested *H. fraxineus* isolates (representing all five populations) were 40.26 mm (95% confidence interval (CI): 34.90, 40.27) during the 3°C growth experiment. This value was nearly identical to that observed in the high‐temperature growth experiment (40.14 mm, CI: 39.12, 41.16). In contrast, isolates in the 6°C growth experiment exhibited a slightly larger mean colony diameter of 43.36 mm (CI: 40.81, 45.92) (Figure 1). Within each experiment, growth estimates at 22°C showed no significant variation among the populations (Figure 2a, left column, Table 2), indicating that the growth reaction norm intercepts were consistent across populations.

**FIGURE 3:**
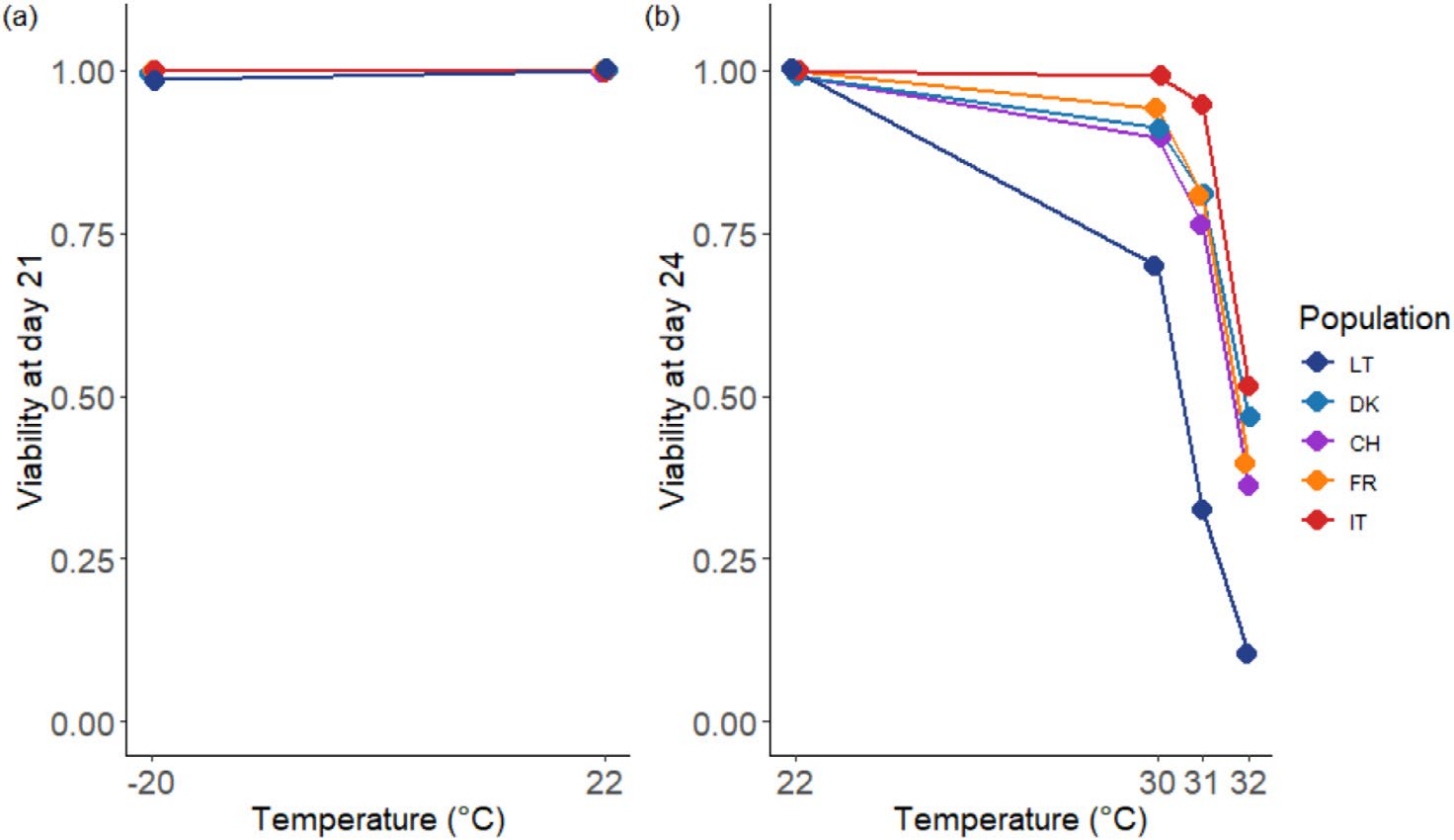
Viability of *Hymenoscyphus fraxineus* isolates following different temperature treatments in two separate experiments at lower (a) and higher (b) temperatures. The viability rate of each isolate ranges from 0 (no agar plugs resumed growth) to 1 (all agar plugs resumed growth). Each data point represents the mean viability rate of 15 fungal isolates within a specific population, with nine replicates per isolate and per temperature. Colors and letters correspond to the origin of the isolates: Dark blue represents Lithuania (LT), blue represents Denmark (DK), purple represents Switzerland (CH), orange represents France (FR), and red represents Italy (IT).

**FIGURE 4:**
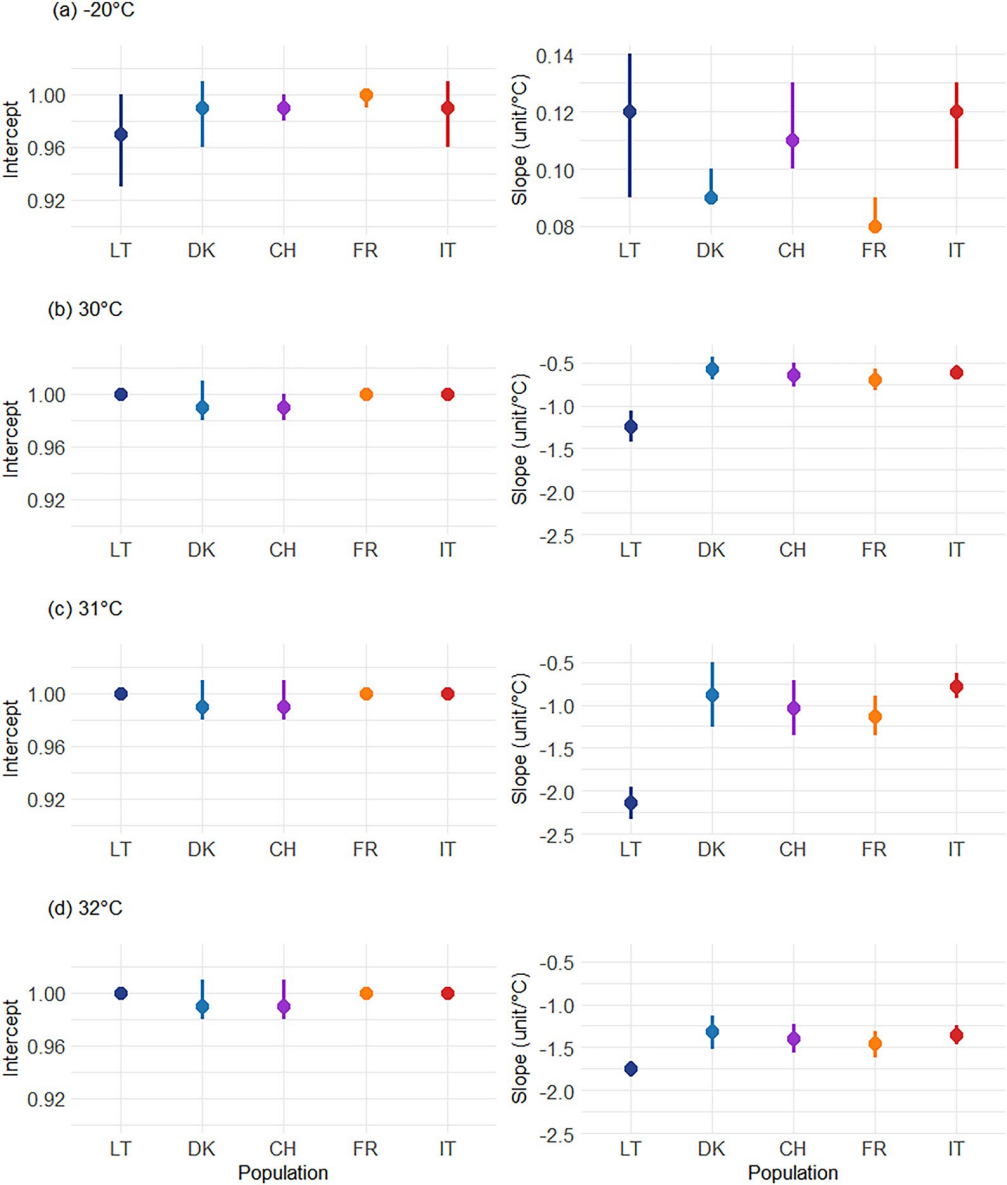
Estimated intercepts and slopes (unit/°C) of viability reaction norms in five European *Hymenoscyphus fraxineus* populations exposed to different temperatures for 21 and 24 days, respectively. Viability ranges from 0 (no agar plugs resumed growth) to 1 (all agar plugs resumed growth). Panels (a) to (d) correspond to the different temperature treatments. The reaction norms were estimated based on individual diameter measurements at 22°C (control temperature) and at the corresponding treatment temperature. Each data point represents the mean of the estimates of viability of 15 fungal isolates within a specific population, with nine replicates per isolate and per temperature. Error bars indicate the 95% confidence interval of the estimates. The colors and letters indicate the origin of the isolate: Dark blue for Lithuania (LT), blue for Denmark (DK), purple for Switzerland (CH), orange for France (FR), and red for Italy (IT).

In both experiments assessing isolate viability, the average viability rate at 22°C ranged from 0.99 to 1 (Figure 3). Likewise, no significant differences were detected in the reaction norm intercept estimates across populations (Figure 4a, left column, Table 2).

### 3.2 Consistent Growth Response to Low‐Temperature Treatments, but Variable Responses to Temperature Increase Across Populations

Low‐temperature treatments (6°C and 3°C) inhibited the diameter growth of *H. fraxineus* cultures across the studied populations. The estimated reaction norms for growth exhibited average slopes of −1.97 mm/°C across the 3°C–22°C interval and −2.34 mm/°C across the 6°C–22°C interval (Figure 2a,b, right column). At 3°C, the mean predicted colony diameters were uniformly zero across all populations (Figure 1 and Figure S1a). At 6°C, the mean predicted colony diameter ranged from 4.91 mm in the Lithuanian population to 6.58 mm in the Swiss population, with no significant differences observed (Figure S1b).

Increasing the temperature from 22°C to 24°C slightly increased the mean colony diameters for all populations (0.56–1.8 mm/°C), except for the Lithuanian population. This population exhibited the lowest reaction norm slope estimate (−0.82 mm/°C; CI: −1.15, −0.49), which significantly differed from the positive slope estimates observed in the other populations (Figure 2c, right column). At 24°C, isolates from the Lithuanian population exhibited a mean colony diameter slightly lower than that of the Danish population (Figure 1b and Figure S1c).

The 26°C treatment resulted in near‐zero reaction norm slope estimates for the French (−1.29 mm/°C, CI: −2.19, −0.38), Danish (−0.96 mm/°C, CI: −1.96, 0.05), Lithuanian (−0.29 mm/°C, CI: −2.71, 2.14), and Swiss (0.15 mm/°C, CI: −0.82, 1.12) populations (Figure 2d, right column). At this temperature, the isolates from these populations exhibited relatively similar colony diameter estimates, ranging from 33.48 mm (French population, CI: 29.22, 37.75) to 42.25 mm (Swiss population, CI: 38.31, 46.2) (Figure 1b and Figure S1d). In contrast, the Italian population showed a steady increase in colony diameter following a temperature increase from 22°C to 26°C (slope: 8.83 mm/°C, CI: 7.21, 10.46; diameter at 26°C: 74.80 mm, CI: 69.43, 80.16), resulting in a statistically significant difference in slope estimate between this population and the others (Figure 2d, right column). Similarly, colony diameter estimates at 26°C were significantly higher for isolates representing the Italian population compared to those of other populations (Figure 1b and Figure S1d).

The 27°C treatment had a consistent and strong effect on colony diameters across populations. The average estimated slope of the reaction norm was −6.59 mm/°C (Figure 2e, right column). However, at 27°C the mean predicted colony diameter of Lithuanian *H. fraxineus* isolates was smaller (2.38 mm, CI: 1.68–3.09) than the mean colony diameters from the other four populations (8.47 mm) (Figure 1b and Figure S1e). Given the uniform growth observed across populations at lower temperatures, this result suggests that the Lithuanian population has the narrowest thermal niche.

### 3.3 Homogeneous Responses of Populations' Viability to Low‐Temperature Treatments, Variable Response to Temperature Increase

Viability rates estimated for *H. fraxineus* isolates exposed to −20°C did not differ significantly from 1 (Figure 3a and Figure S2a), indicating good tolerance to subzero temperatures by the fungus. The reduction in individual viability rates across the −20°C to 22°C temperature range was minimal (0.08–0.12 units/°C), with significant yet marginal differences among populations (Figure 4a, right column).

In contrast, high‐temperature treatments had a strong but relatively uniform effect on the viability of Danish, Swiss, French, and Italian populations. For isolates from these populations, the average decline in viability was −0.63, −0.96, and −1.38 units/°C under the 30°C, 31°C, and 32°C treatments, respectively (Figure 4b–d, right column). Notably, Lithuanian isolates exhibited significantly steeper reaction norm slopes estimated across all three treatments, with declines of −1.24 units/°C at 30°C, −2.14 units/°C at 31°C, and −1.75 units/°C at 32°C (Figure 4b–d, right column).

Due to distinct plastic responses, the predicted mean viability rate of the Lithuanian isolates at 30°C (0.71, CI: 0.55–0.87) was significantly lower than that of isolates from the southernmost Italian population (0.99, CI: 0.98–1.01) (Figure S2b). This disparity became even more pronounced at 31°C, where Lithuanian isolates exhibited the lowest viability rate (0.33) compared to the mean predicted viability rate of the other populations (0.84) (Figure S2c). At 32°C, Lithuanian isolates showed the lowest viability rate (0.11), whereas isolates from the other four populations displayed viability rates approximately four times higher (Figure S2d).

### 3.4 Variation in Maximal Colony Diameters and Optimal Temperature for Growth Among the Populations

The maximal mean colony diameters for isolates from each population were observed within the temperature range of 22°C–26°C. The growth plasticity of the isolates from the Lithuanian, Danish, Swiss, and French *H. fraxineus* isolates was relatively low between 22°C and 26°C (see above), resulting in consistent colony diameters across this temperature range and thus indicating no clear optimal temperature for mycelial growth across this temperature interval. In contrast, the Italian population displayed a clear temperature‐dependent growth pattern, reaching the largest colony diameter at 26°C (74.80 mm, CI: 69.43, 80.16), compared to 39.37 mm (CI: 35.19, 43.55) at 22°C and 41.85 mm (CI: 37.84, 45.87) at 24°C Figure 1b.

The maximal colony sizes observed over the tested temperature range were similar for the French (42.84 mm, CI: 39.38–46.31), Swiss (50.36 mm, CI: 43.59–57.13), and Danish isolates (46.11 mm, CI: 41.44–50.77) (Figure 1b). Lithuanian isolates reached a mean maximal colony diameter of 39.10 mm (CI: 35.85–42.36), which was comparable to those of the French and Swiss isolates but smaller than that of the Danish isolates. In contrast, Italian isolates exhibited a significantly larger mean maximal colony diameter, reaching 74.80 mm (CI: 69.43–80.16).

### 3.5 Differences in Within‐Population Variability of Growth Reaction Norms, No Difference for Viability

In both the 3°C–22°C and 6°C–22°C temperature ranges, *H. fraxineus* isolates representing the French and Italian populations consistently exhibited lower variability in both the intercept (i.e., colony diameter growth at 22°C) and slopes of the growth reaction norm. This trend was particularly pronounced for the French isolates (Figures S3 and S4a,b). Across these temperature intervals, the Swiss isolate showed the most variable growth reaction norms. Few differences in growth reaction norm variability were observed in the 22°C–24°C temperature range across populations (Figure S3c). Similarly, the variability in the intercepts of the growth reaction norms was homogeneous in the 22°C–26°C and 22°C–27°C temperature ranges (Figure S3d,e). However, some differences in the variability of growth plasticity were detected, depending on the population. For example, in the 22°C–26°C temperature range, Lithuanian and Italian isolates exhibited more than double the slope variability of the other populations (Figure S3d, right column). Likewise, in the 22°C–27°C range, Italian and Swiss isolates displayed slightly greater slope variability compared to French and Lithuanian isolates (Figure S3e, right column).

No significant differences in reaction norm variability for isolate viability were observed among the *H. fraxineus* populations studied (Figures S5 and S6).

## 4 Discussion

Evaluating the influence of phenotypic plasticity on population expansions in spatially heterogeneous landscapes is necessary for predicting future distributions of species. A first step in this direction is to determine whether and how the plasticity of adaptive traits itself is evolving. Ash dieback in Europe serves as an emblematic example of a forest epidemic caused by a nonnative fungal pathogen. The ongoing expansion of ash dieback along a north–south gradient offers a unique opportunity to test theoretical predictions about the evolution of phenotypic plasticity in relation to temperature in *H. fraxineus* while also informing predictive models and disease management plans. To the best of our knowledge, this is the first study of this kind aiming at characterizing the evolution of thermal reaction norms in an invasive forest fungal pathogen on a Europe‐wide scale.

In our experiments, the response of isolates from five European *H. fraxineus* populations to low‐temperature treatments showed consistent patterns, with in vitro mycelial growth exhibiting a globally uniform reduction. Similarly, the viability of isolates from European populations following treatment with negative temperatures remained remarkably stable across all populations. In contrast, the investigated populations differed in their responses to high temperatures. Interestingly, the isolates from the Italian population exhibited both the highest optimal growth temperature (26°C vs. 22°C to 24°C optimal for the other herein‐investigated *H. fraxineus* populations) and the highest mean growth rate. In addition, the growth and viability of Lithuanian isolates were the most negatively affected by high temperatures, suggesting that this population has the narrowest thermal niche breadth. Furthermore, we observed that within‐population variability in the slopes of individual reaction norms for mycelial growth differed among populations. Notably, this variability was highest under the high‐temperature treatment in the Lithuanian, Swiss, and Italian populations. These findings support the hypothesis that *H. fraxineus* plasticity in growth and viability has evolved during the spread of the ash dieback epidemic across Europe. Here, we discuss the overall pattern of reaction norms observed, followed by the evolutionary processes that may have driven divergences among the populations.

### 4.1 Comparison With Reaction Norms Previously Characterized in *H. fraxineus*

The present study showed that *H. fraxineus* cultures remained viable across a temperature range from −20°C to 32°C. The fungus was able to grow between 6°C and 27°C, with optimal growth between 22°C and 26°C. These reaction norms are consistent with those of mesophilic fungi, which typically grow between 0°C and 50°C, with optimal growth temperatures ranging between 15°C and 40°C (Dix and Webster 1995).

Our findings are in good agreement with earlier studies by Kowalski and Bartnik (2010) and Hauptman et al. (2013), which focused on the performance of *H. fraxineus* populations from Poland and Slovenia, respectively. Yet, the growth rates of our populations were generally higher than those reported by these earlier studies. For instance, in our experiments, the average growth rate at 22°C over a two‐week period was 2.87 mm/day, compared to 1.60 mm/day at 20°C and 1.74 mm/day at 22°C, as reported by Kowalski and Bartnik (2010) and Hauptman et al. (2013), respectively. These discrepancies may stem from variations in the culture media used. Indeed, the AMEA medium used in our experiments is reported to support better or faster growth of *H. fraxineus* than most other commonly used media (Botella et al. 2016; Kirisits et al. 2013). Additionally, differences in the duration of temperature exposure may have contributed to discrepancies observed (two weeks in the present study vs. five weeks in the above‐mentioned studies) (Abu Bakar et al. 2020). The study by Hauptman et al. (2013) is the only study we found addressing the viability of *H. fraxineus* in relation to temperature. However, the viability of *H. fraxineus* was assessed in ash saplings and shoots under high‐temperature treatments, which compromises direct comparison with our results.

### 4.2 Contrasting Balances Between Genetic Drift and Selection

Earlier studies indicated that European populations of *H. fraxineus* were most likely composed of descendants of two parental strains that were genetically highly divergent (Gross, Hosoya, and Queloz 2014; McMullan et al. 2018). Following the strong bottleneck undergone by this sexually reproducing fungus, a high genotypic diversity was observed within established populations, which showed no genetic differentiation (Burokiene et al. 2015; Gross, Hosoya, and Queloz 2014; McMullan et al. 2018). These results might point to a low evolutionary potential of the pathogen. Nonetheless, the above‐mentioned studies were carried out at an anterior stage of the epidemic development, and none of them have considered the southernmost *H. fraxineus* populations included in our experimental setting. We discuss here the evolutionary processes that may have shaped the differentiation in thermal plasticity observed among the sampled populations.

Genetic drift, defined as random changes in genetic variation from generation to generation, may contribute to phenotypic divergence among populations (Lynch 2007). The impact of genetic drift on evolutionary trajectories is amplified by strong demographic fluctuations (Hermisson and Pennings 2005), which are particularly likely to occur at the leading edge of a species' distribution range (Wright 1982). Since phenotypic plasticity is considered an evolving trait (Ghalambor et al. 2007), the divergences in reaction norms for growth and viability observed in our study could potentially be attributed to genetic drift. On the other hand, latitudinal variations in temperature conditions may expose European *H. fraxineus* populations to divergent selection pressures (Booker 2024; Schmid and Guillaume 2017). Moreover, substantial gene flow likely interconnects European *H. fraxineus* populations (Fones et al. 2016), thereby acting as a buffer against random genetic drift. In such conditions, natural selection may primarily drive reaction norm differentiation across large heterogeneous landscapes (Whitlock 2004).

The northernmost (Lithuanian) and the southernmost (Italian) *H. fraxineus* populations consistently exhibited the greatest divergence in evolutionary trajectories in both growth and viability traits (Lithuania) or solely in growth traits (Italy). Notably, these two populations also displayed the highest levels of within‐population variability in reaction norms for growth, which speaks against the hypothesis that genetic drift alone is responsible for the observed divergences. Assuming that higher mycelium growth rates confer increased fitness to the fungus (Blenis et al. 1984; Kowalski and Bartnik 2010), the shifts observed in both the plasticity of growth and the optimal growth temperature in *H. fraxineus* isolates of the Italian population, aligning with the north–south temperature gradient, strongly support the adaptationist hypothesis. This hypothesis is further supported by a similar pattern of divergence observed for viability and its plasticity between the Lithuanian population (the northernmost and thus exposed to the lowest temperatures) and other investigated populations.

The increased growth plasticity observed in the Italian population, particularly across the 22°C–26°C temperature interval, aligns with theoretical predictions that plasticity can facilitate population survival in rapidly changing environmental conditions (Fox et al. 2019; Lande 2009, 2015; Reed et al. 2010; Scheiner et al. 2022). Furthermore, our results are consistent with the predictions of Ruiz‐Aravena et al. (2014), which suggest that directional selection induced by recurrent high temperatures can increase the maximum temperature at which growth and survival are possible, without altering the minimum temperature required for sufficient trait expression. Additionally, the observed patterns of divergence align with the theory about plasticity evolution, indicating that thermal niches may evolve via a “sidestep niche” model. This model describes a niche transformation characterized by an initial expansion, followed by a shift, and ultimately a contraction (Gallet et al. 2014; Lande 2009; Lázaro‐Nogal et al. 2015).

### 4.3 Other Possible Influences

Unlike the Italian population, the French population did not express reaction norms for growth and viability differing from those of the Swiss and Danish populations, despite being exposed to the highest mean annual temperatures (Table 1). Based on the earliest local disease reports from the sampling sites, the French *H. fraxineus* population appears to be the youngest among those studied (Table 1). This raises the assumption that natural selection has not yet had sufficient time to induce phenotypic changes. Below we discuss four additional factors that may have interacted with the temperature gradient and influenced the evolution of thermal plasticity near the epidemic front.

- Humidity. The climatic conditions at the French site were markedly drier than at the Italian site (Table 1). Since air humidity is known to promote fungal development (Burdon 1991), these climatic differences may have influenced the effective population sizes (Grosdidier et al. 2020). A possible low effective population size in the French *H. fraxineus* population may have constrained its adaptation to high temperatures.
- Composition of forest stands. Differences in ash stand density among sites may have influenced the adaptive response of *H. fraxineus* populations by determining their effective size (Laubray et al. 2023). In addition, susceptibility to dieback varies significantly both within *F. excelsior* (McKinney et al. 2014; Lobo et al. 2015; Doonan et al. 2025) and across ash species (Gross and Sieber 2016; Schwanda and Kirisits 2016; Adamčíková et al. 2018). This susceptibility is heritable (Lobo et al. 2015; Doonan et al. 2025) and appears to be determined by plant traits such as phenolic compound production (Villari et al. 2018) or bud burst phenology (Stener 2013). Importantly, variability in dieback susceptibility may influence both the demography of *H. fraxineus* populations and the evolution of life‐history traits related to disease expression (Thrall and Burdon 2003; Papaïx et al. 2018), including mycelial growth (Blenis et al. 1984; Kowalski and Bartnik 2010). To our knowledge, no large‐scale spatial structure in dieback susceptibility has been reported for *F. excelsior* across Europe. However, differences in ash species composition were observed among the study sites. Specifically, *F. excelsior* (highly susceptible to the disease (Gross and Sieber 2016)) is more common in southern France than in northern Italy (Beck et al. 2016), while *F. ornus* (a species highly resistant to the disease) is much more prevalent in northern Italy than in southern France (Caudullo and De Rigo 2016). This suggests that *H. fraxineus* may have experienced stronger selective pressures in northern Italy than in southern France, which potentially contributed to the observed differences in thermal plasticity of mycelial growth of the fungus.
- Micro‐environmental variations. Within each site, temperature and humidity may vary locally due to differences in sun exposure, proximity to water bodies, or elevation. Likewise, the spatio‐temporal distribution of ash resistance might be strongly site‐specific. These micro‐environmental variations could interact with broader‐scale patterns of variation in climate, host availability, and host resistance, potentially influencing the evolution of thermal reaction norms (Bacigalupe et al. 2018; Vinton et al. 2022; Usui et al. 2023).
- Secondary introduction. Previous studies on the genetic composition of European *H. fraxineus* populations were conducted prior to the first reports of ash dieback in southern France and northern Italy. The Italian population may result from a recent introduction of novel *H. fraxineus* genotypes. Such an introduction could have enabled a rapid response to local selective pressures, fostering both phenotypic and genetic divergence from other populations (McMullan et al. 2018).

## 5 Concluding Remarks

We observed that both the plasticity of mycelial growth and the temperature sensitivity of viability have evolved in European epidemic and post‐epidemic populations of *H. fraxineus*. Our study underscores the need for further investigation, including the influence of other climatic factors, such as humidity, and the role of additional adaptive traits at different stages of the fungal life cycle, such as gamete production, aggressiveness, and morphotype variation. Given the ongoing epidemic dynamics, future studies characterizing a broader range of thermal reaction norms across finer temperature intervals would also be valuable for confirming and further elucidating the evolutionary trends identified here. For this purpose, accurate information about the biotic and abiotic environmental conditions at each sampling site would help to further interpret the identified trends. Analyses of the genetic composition of the population and how this relates to the phenotypic variation observed here will also be essential. Specifically, such analyses may be helpful in determining whether the Italian *H. fraxineus* population results from a secondary introduction, which could explain the differentiation in the reaction norms observed here.

Our results confirm that the understanding of pathogen evolution is crucial for the prediction of disease risk in forest ecosystems and for developing effective control strategies (Desprez‐Loustau et al. 2016). Specifically, integrating the phenotypic plasticity of pathogen traits and their evolution into long‐term disease risk models (Desprezloustau et al. 2007; Goberville et al. 2016) could improve prediction accuracy and support the design of effective control measures.

Overall, our findings suggest that the risk of ash dieback may be considerably higher than expected in warm and humid areas along the epidemic front (Marçais et al. 2023). A sustainable strategy for forest restoration and ash preservation in vulnerable sites could involve locally mixing ash with non‐host tree species. This approach would leverage a dilution effect preventing *H. fraxineus* transmission and evolution (Keesing and Ostfeld 2021). The use of resistant ash plants selected in current breeding programs may also help constrain disease development. However, particular attention should be paid to the preservation of existing genetic diversity in European ash (McKinney et al. 2014; George et al. 2024). Indeed, reduced genetic diversity within ash populations could trigger adaptive responses in *H. fraxineus* populations (Papaïx et al. 2018) or further increase the vulnerability of ash trees to other pests or extreme climatic events (Climent et al. 2024).

## Author Contributions

**Clémence Bécans:** conceptualization (lead), formal analysis (lead), investigation (lead), methodology (lead), resources (lead), software (supporting), validation (lead), visualization (lead), writing – original draft (lead), writing – review and editing (supporting). **Cécile Robin:** conceptualization (lead), investigation (supporting), methodology (lead), project administration (supporting), writing – original draft (supporting), writing – review and editing (supporting). **Katharina B. Budde:** resources (supporting), writing – original draft (supporting), writing – review and editing (supporting). **Luisa Ghelardini:** resources (supporting), writing – original draft (supporting). **Andrin Gross:** resources (supporting), writing – original draft (supporting). **Vaidotas Lygis:** resources (supporting), writing – original draft (supporting), writing – review and editing (supporting). **Lene Rostgaard Nielsen:** resources (supporting), writing – original draft (supporting). **Gilles Saint‐Jean:** investigation (supporting), resources (supporting). **Jean‐Paul Soularue:** conceptualization (lead), formal analysis (lead), investigation (lead), methodology (lead), project administration (lead), resources (lead), software (lead), supervision (lead), validation (lead), visualization (lead), writing – original draft (lead), writing – review and editing (lead).

## Conflicts of Interest

The authors declare no conflicts of interest.

## Supporting information


Figure S1.–S6.

## Data Availability

The data and code supporting the findings of this study are openly avalaible at https://doi.org/10.57745/MKXIYI (Bécans and Soularue 2025).
