# Supplementary material for: Evolution of Thermal Plasticity in Hymenoscyphus fraxineus During Ash Dieback Expansion in Europe
Source: Ecol Evol. 2025 Jun 17;15(6):e71513. doi: 10.1002/ece3.71513 (PMC12173838; doi:10.1002/ece3.71513)
Supplement: Supplementary file 1 — Figure S1.–S6. [file ECE3-15-e71513-s001.docx]

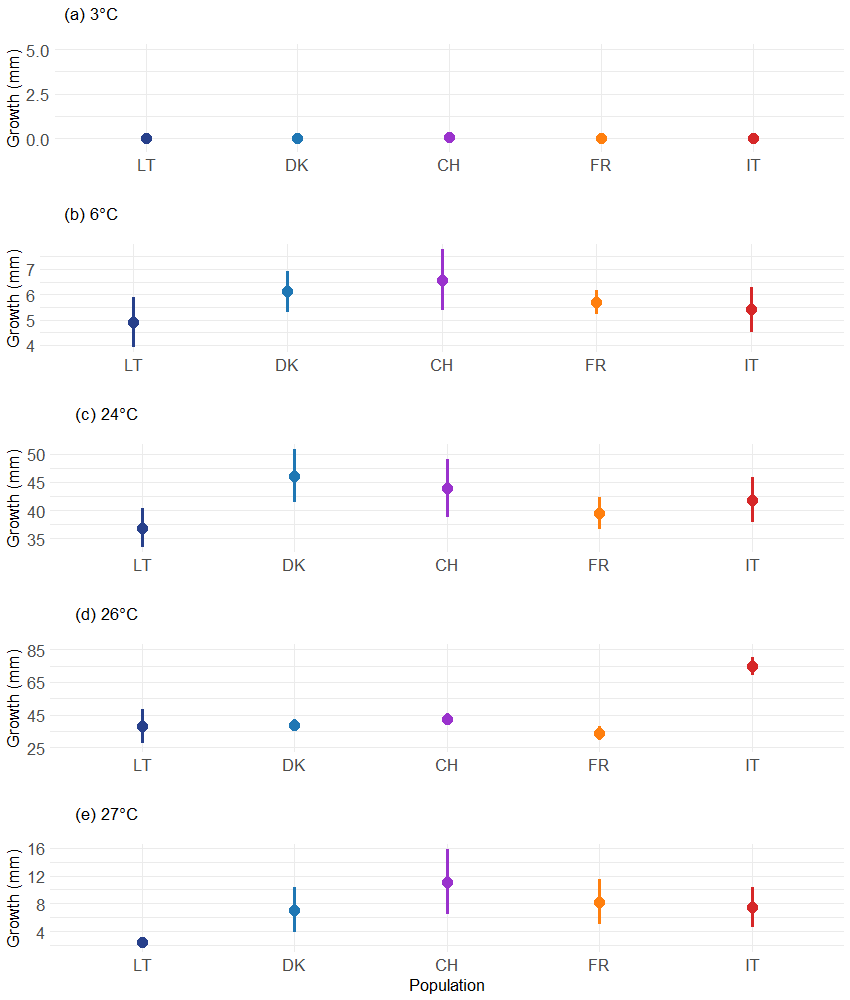


**Figure S1.** Predictions of 14-day-old mycelial growth (mm) in five European *Hymenoscyphus fraxineus* populations cultured at different temperatures. Panels (a) to (d) refer to the different temperature treatments. Each data point represents the mean of the culture diameter estimates (in mm) of 15 fungal isolates within a specific population, with three replicates per isolate and per temperature. Error bars indicate the 95% confidence interval of the estimates. The colors and letters indicate the origin of the isolate: dark blue for Lithuania (LT), blue for Denmark (DK), purple for Switzerland (CH), orange for France (FR), and red for Italy (IT).


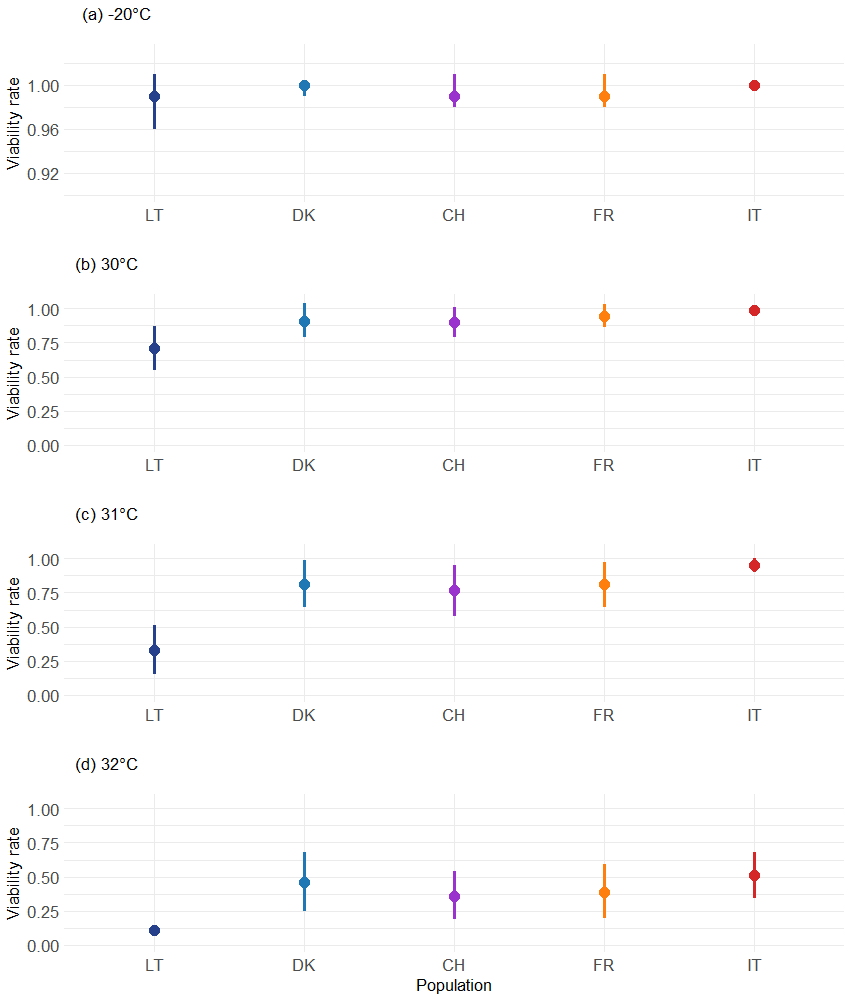
**Figure S2.** Predictions of 21 and 24-days viability of *Hymenoscyphus fraxineus* isolates from five European populations, cultured at four different temperatures. Viability ranges from 0 (no agar plugs resumed growth) to 1 (all agar plugs resumed growth). Panels (a) to (d) refer to the different temperature treatments. Each data point represents the mean of the viability estimates of 15 fungal isolates within a specific population, with nine replicates per isolate and per temperature. Error bars indicate the 95% confidence interval of the estimates. The colors and letters indicate the origin of the isolate: dark blue for Lithuania (LT), blue for Denmark (DK), purple for Switzerland (CH), orange for France (FR), and red for Italy (IT).


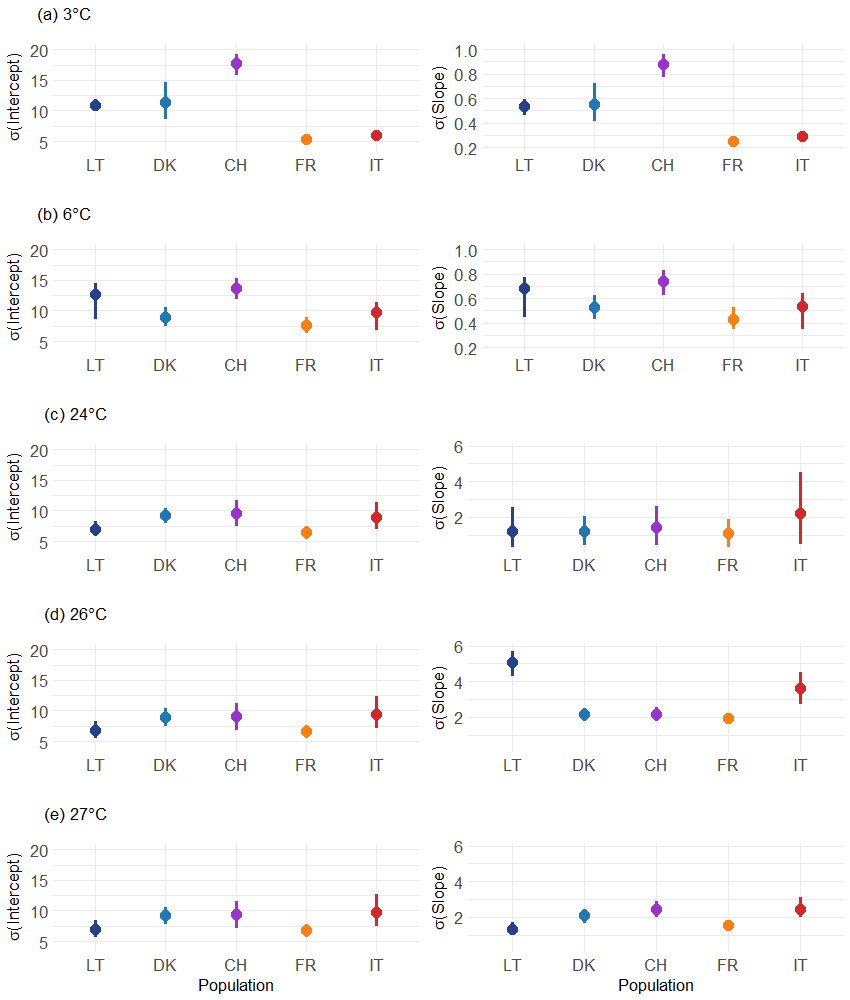


**Figure S3.** Estimated variability of intercepts (mm) and slopes (mm/°C) of growth reaction norms in five European *Hymenoscyphus fraxineus* populations exposed to different temperatures for two weeks. Panels (a) to (e) refer to the different temperature treatments considered. Each data point represents the mean of the parameter estimates of 15 fungal isolates within a specific population, with three replicates per isolate and per temperature. Error bars indicate the 95% confidence interval of the estimates. The colors and letters indicate the origin of the isolate: dark blue for Lithuania (LT), blue for Denmark (DK), purple for Switzerland (CH), orange for France (FR), and red for Italy (IT).


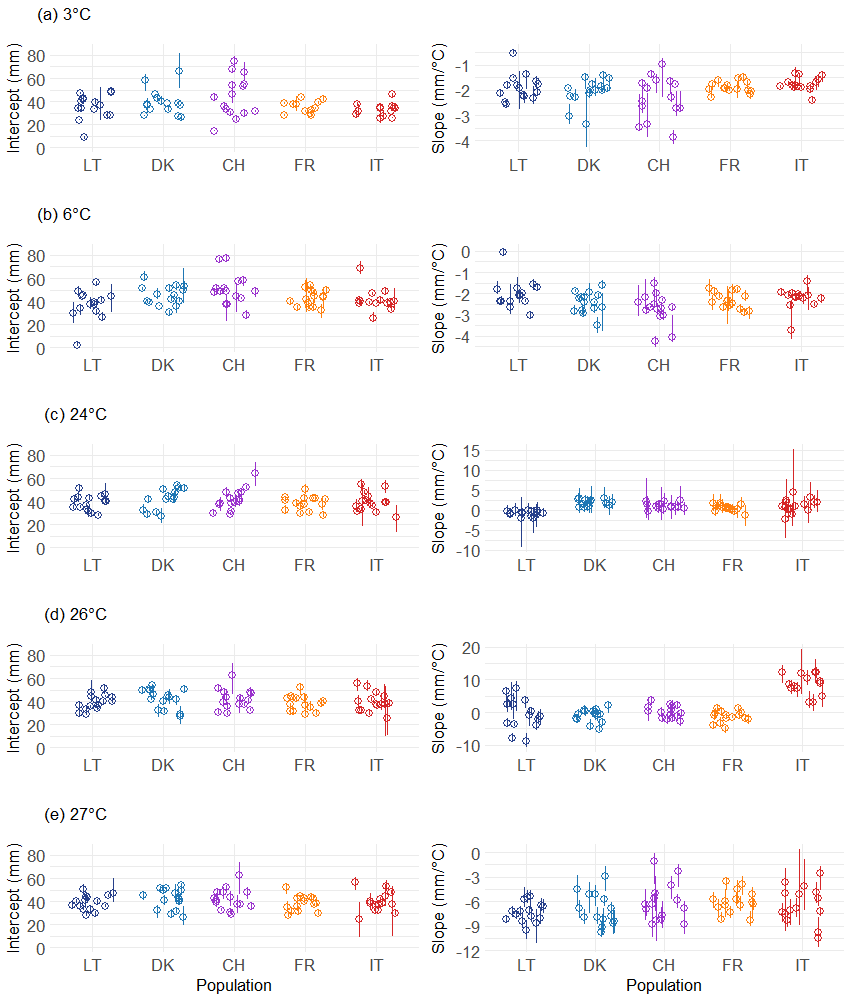
**Figure S4**. Estimated intercepts (mm) and slopes (mm/°C) of individual growth reaction norms in five European *Hymenoscyphus fraxineus* populations following a two-week exposure of the fungal isolates to different temperatures Panels (a) to (e) refer to the different temperature treatments considered. Each data point represents the mean parameter estimate of 15 fungal isolates within a specific population, with three replicates per isolate and per temperature. Error bars indicate the 95% confidence interval of the estimates. The colors and letters indicate the origin of the isolate: dark blue for Lithuania (LT), blue for Denmark (DK), purple for Switzerland (CH), orange for France (FR), and red for Italy (IT).


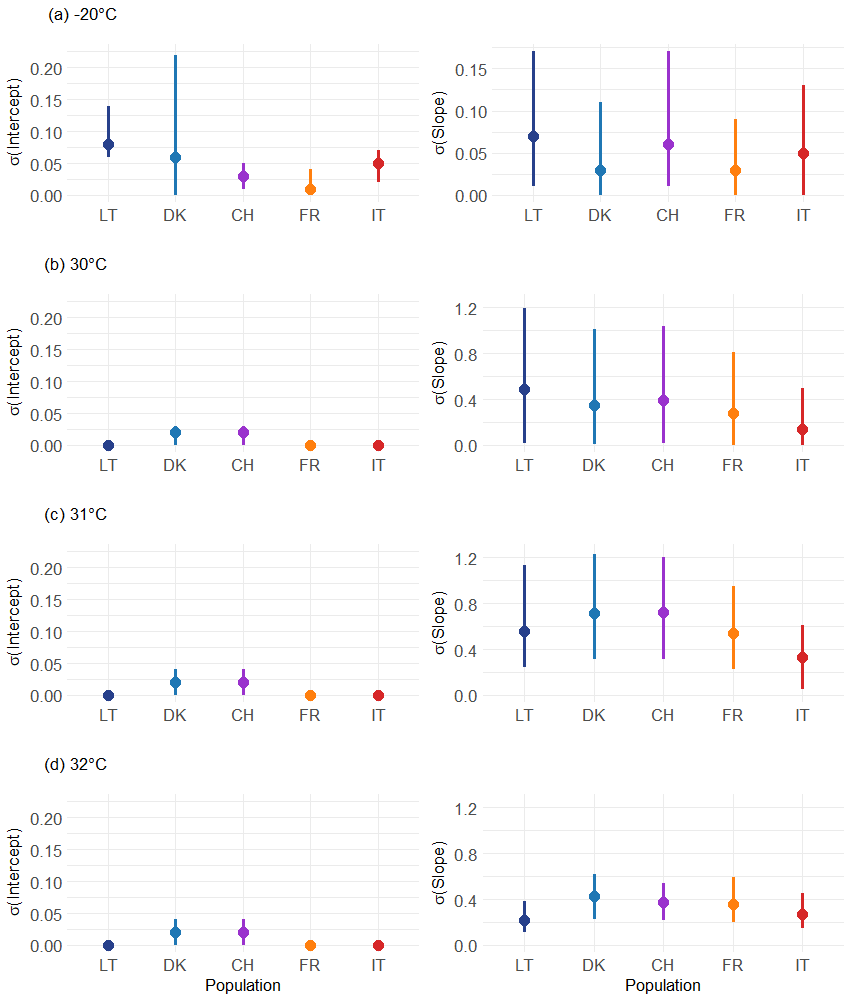


**Figure S5**. Estimated variability of intercepts and slopes (unit/°C) of viability reaction norms in five European *Hymenoscyphus fraxineus* populations exposed to different temperatures for 21 and 24 days respectively. Viability ranges from 0 (no agar plugs resumed growth) to 1 (all agar plugs resumed growth). Panels (a) to (d) refer to the different temperature treatments considered. Each data point represents the mean of the parameter estimates of 15 fungal isolates within a specific population, with nine replicates per isolate and per temperature. Error bars indicate the 95% confidence interval of the estimates. The colors and letters indicate the origin of the isolate: dark blue for Lithuania (LT), blue for Denmark (DK), purple for Switzerland (CH), orange for France (FR), and red for Italy (IT).


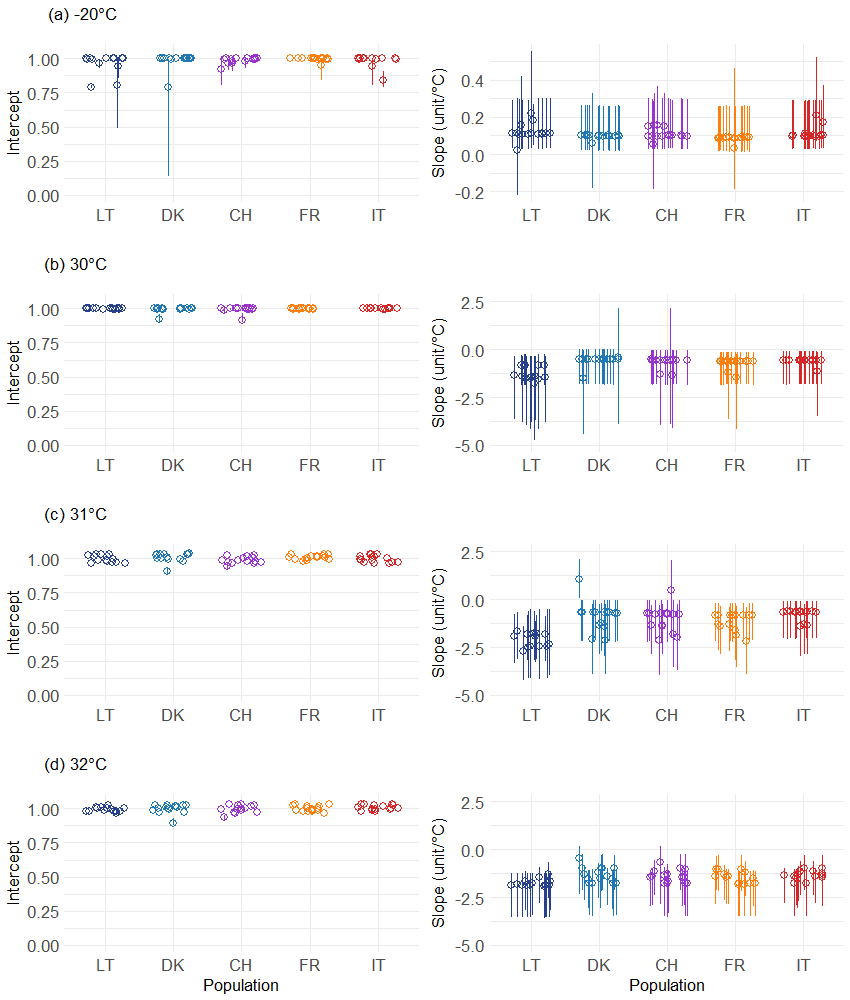


**Figure S6.** Estimated intercepts and slopes (unit/°C) of individual viability reaction norms in five European *Hymenoscyphus fraxineus* populations following 21- and 24-day exposure of the fungal isolates to different temperatures. Viability ranges from 0 (no agar plugs resumed growth) to 1 (all agar plugs resumed growth). Panels (a) to (d) refer to the different temperature treatments considered. Each data point represents the mean parameter estimate of 15 fungal isolates within a specific population, with nine replicates per isolate and per temperature. Error bars indicate the 95% confidence interval of the estimates. The colors and letters indicate the origin of the isolate: dark blue for Lithuania (LT), blue for Denmark (DK), purple for Switzerland (CH), orange for France (FR), and red for Italy (IT).
